# Therapeutic Effect of Ecdysterone Combine Paeonol Oral Cavity Direct Administered on Radiation-Induced Oral Mucositis in Rats

**DOI:** 10.3390/ijms20153800

**Published:** 2019-08-03

**Authors:** Li Yang, Jian Pan

**Affiliations:** School of Food and Biological Engineering, Hefei University of Technology, Hefei 230009, China

**Keywords:** therapeutic effect, radiation-induced oral mucositis, ecdysterone-paeonol, oral cavity direct administered, compound-target network, autodock

## Abstract

Radiation-induced oral mucositis represents an influential factor in cancer patients’ accepted radiation therapy, especially in head and neck cancer. This research investigates the treatment effect of Ecdysterone (a steroid derived from the dry root of *Achyranthes bidentate*) and Paeonol (a compound derived from *Cortex Moutan*) on radiation-induced oral mucositis and possible underlying mechanisms. Concisely, 20 Gy of X-rays (single-dose) irradiated the cranial localization in rats for the modeling of oral mucositis. The therapeutic effects of Ecdysterone-Paeonol oral cavity directly administered on radiation-induced oral mucositis were investigated by weight changes, direct observations, visual scoring methods, ulcer area/total area, and basic recovery days. Assessments of tumor necrosis factor α and interleukin-6 were performed to evaluate the inflammatory cytokines secretion in the damaged areas of tongues harvested post-treatment, and changes in signaling pathways were investigated by Western blotting. System Drug Target (SysDT) methods revealed the targets of Ecdysterone-Paeonol in order to support compound-target network construction. Four representative targets with different functions were chosen. The binding interactions between the compound and receptor were evaluated by molecular docking to investigate the binding affinity of the ligand to their protein targets. Ecdysterone-Paeonol, administered orally, effectively improved radiation-induced oral mucositis in rats, and the therapeutic effect was better than Ecdysterone administered orally on its own. In this study, calculational chemistry revealed that Ecdysterone-Paeonol affected 19 function targets associated with radiation-induced oral mucositis, including apoptosis, proliferation, inflammation, and wound healing. These findings position Ecdysterone-Paeonol as a potential treatment candidate for oral mucositis acting on multiple targets in the clinic.

## Significance Statement

Radiation-induced oral mucositis (RIOM), a normal oral cavity tissue injury resulting from radiotherapy, represents an influential factor in cancer patients’ accepted radiation therapy, especially in head and neck cancer patients. Quality of life, economic cost, and radiotherapy continuity are affected by RIOM, which slows the cancer therapy and impoverishes tumor control.

In this work, we uncover the therapeutic effect of Ecdysterone combine Paeonol on radiation-induced oral mucositis. The Ecdysterone-Paeonol combination drug reduces pro-inflammatory cytokines production by inhibiting the NF-κB pathway. Systems pharmacology dissection results uncovered the detailed molecular mechanism and the multiple targets of Ecdysterone-Paeonol. These findings position Ecdysterone-Paeonol as a potential treatment candidate.

## 1. Introduction

Radiation-induced oral mucositis (RIOM), a normal oral cavity tissue injury resulting from radiotherapy, represents an influential factor in cancer patients’ accepted radiation therapy, especially in head and neck cancer patients [1,2,3]. Quality of life, economic cost, and radiotherapy continuity are affected by RIOM, which slows the cancer therapy and impoverishes tumor control [4]. Unfortunately, RIOM, throughout the whole course of radiotherapy, is a huge challenge for radiation oncologists, like climbing an endless mountain [5].

Recent studies have proposed that radiation-induced oral mucositis involves five representative biologic progression phases [3,6]: Phases I, initiation stage characterized by large reactive oxygen species (ROS) production; Phases II/Ⅲ, message generation/signaling and amplification stage characterized by large epithelial ablation caused by mass-apoptosis and halted cell proliferation; Phases IV, ulceration stage characterized by a large area of mucositis and pro-inflammatory cytokines secretion; and Phases V, healing stage characterized by slow wound healing. As we can see, apoptosis, proliferation, inflammation, and wound healing present different functions in the pathological development course of RIOM.

**Figure 1:**
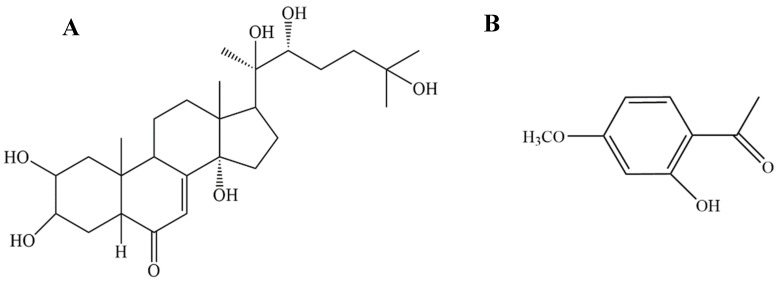
Chemical structures of Ecdysterone and Paeonol: (**A**) Ecdysterone is a steroid derived from *Achyranthes bidentata*, a species of plant in the amaranth family; (**B**) Paeonol is a compound derived from *Cortex Moutan*, a species of plant in the Ranunculaceae family.

A previous study by our research group revealed that Ecdysterone (a steroid derived from the dry root of *Achyranthes bidentate*) (Figure 1A) accelerates the healing of radiation-induced oral mucositis in rats by increasing matrix cell proliferation [7]. However, after deeper research, we found that, even though Ecdysterone has an anti-inflammatory effect, the effect is only weak. Pro-inflammatory cytokines, such as TNF-α and IL-6, were lower than in the modeling rats, but there was no significantly difference. As the research went deeper, we found that the weak anti-inflammatory effect of Ecdysterone is a drawback in the course of treatment. Therefore, in order to increase the therapeutic effect, a suitable anti-inflammatory drug had to be used as a supplement.

Consequently, we hypothesized that a combination drug composed of an anti-inflammatory drug and Ecdysterone could increase the therapeutic effect. In the previous study, we used the oral direct administration method. Therefore, the combination anti-inflammatory drug had to meet the same conditions for better transdermal absorption. After preliminary screening, we found that Paeonol (Figure 1B), a compound derived from *Cortex Moutan*, which has been used for its anti-inflammatory, antioxidant, and antibacterial properties, could suit the supplement role [8,9,10,11].

**Figure 2:**
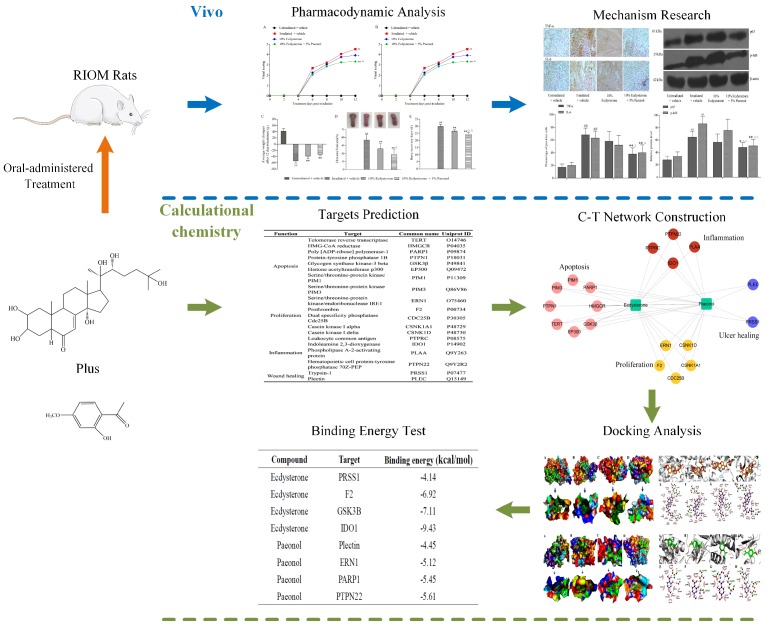
Research scheme for therapeutic effect and possible underlying mechanisms investigation.

In order to uncover the therapeutic effect and possible underlying mechanisms, we performed a systematic research approach as shown in Figure 2. In this study, we demonstrate that Ecdysterone-Paeonol combination drug administered orally effectively improves radiation-induced oral mucositis in rats, and the therapeutic effect is better than Ecdysterone administered orally on its own. A systems pharmacology approach uncovered the pharmacological characteristics of multiple targets, network pharmacology, and molecular docking between Ecdysterone-Paeonol and their receptors. These findings position Ecdysterone-Paeonol as a potential treatment candidate for oral mucositis, acting on multiple targets in the clinic.

## 2. Results

### 2.1. Ecdysterone-Paeonol Alleviates the Development of Radiation-Induced Oral Mucositis, and the Treatment Effect is Better than Ecdysterone Treatment Alone

**Figure 3:**
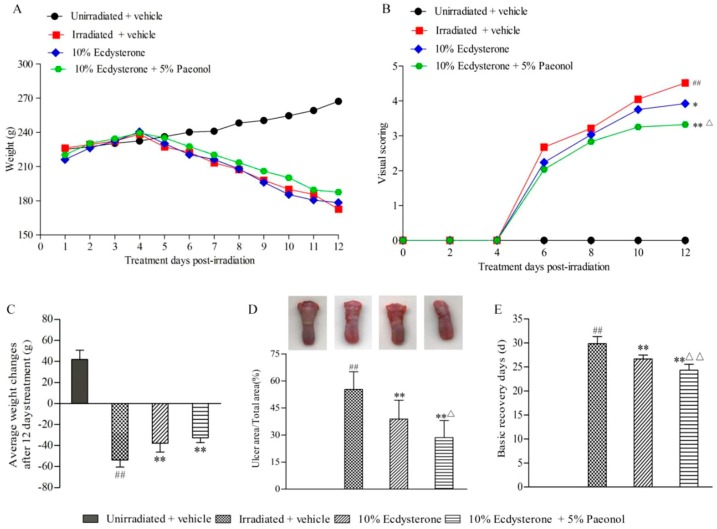
Development of oral mucositis following 12 days of treatment, and basic recovery days after the whole treatment: (**A**) Daily weights (g) for the unirradiated + vehicle, irradiation + vehicle, 10% Ecdysterone, and 10% Ecdysterone + 5% Paeonol groups are shown throughout 12 days of treatment, post-irradiation; (**B**) Average visual scoring (*n* = 6 rats) are shown for every 2nd day throughout 12 days of treatment, post-irradiation (^##^*p* < 0.01 vs. the unirradiated + vehicle group; **p < 0.01, *p < 0.05 vs. the irradiated + vehicle group; ^△^*p* < 0.05 vs. 10% Ecdysterone group after 12 days of treatment; *n* = 6 rats); (**C**) Average weight changes after 12 days of treatment post-irradiation (^##^*p* < 0.01 vs. the unirradiated + vehicle group; **p < 0.01 vs. the irradiated + vehicle group; *n* = 6 rats); (**D**) Representative images and a figure showing the ratio (%) of ulcer area/total area after 12 days of treatment. The unirradiated + vehicle, irradiated + vehicle, 10% Ecdysterone, and 10% Ecdysterone + 5% Paeonol groups are shown (below) (^##^*p* < 0.01 vs. the unirradiated + vehicle group; **p < 0.01 vs. the irradiated + vehicle group; ^△^*p* < 0.05 vs. the 10% Ecdysterone group; *n* = 6 rats). (**E**) Basic recovery days after the whole treatment of each group. (^##^*p* < 0.01 vs. the unirradiated + vehicle group; **p < 0.01 vs. the irradiated + vehicle group; ^△△^*p* < 0.01 vs. the 10% Ecdysterone group; *n* = 6 rats).

After cranial irradiation, the average weight of the irradiated groups began to decline on the fifth day. The decreasing trend of each irradiated group was similar (Figure 3A). However, actual weight loss in the 10% Ecdysterone and 10% Ecdysterone + 5% Paeonol groups was less than in the irradiated + vehicle group (Figure 3C). This indicated that there was a decrease in the RIOM affected rats’ eating desire in each irradiated group, but the situation was improved by treatment with different drugs.

Visual scoring observations every 2nd day throughout the course of treatment revealed that the development of RIOM was alleviated in each treatment group, and the effect in the 10% Ecdysterone + 5% Paeonol group was better than in the 10% Ecdysterone group after 12 days of treatment (Figure 3B). Direct observations and ulcer area also evidenced this result (Figure 3D).

According to the “visual scoring assessment of oral mucositis”, the number of basic recovery days in the treatment group was less than in the irradiated + vehicle group. Better yet, the recovery time of the 10% Ecdysterone + 5% Paeonol group was shorter than the 10% Ecdysterone group (Figure 3E).

All of these results demonstrate that the Ecdysterone-Paeonol combination drug alleviates the development of radiation-induced oral mucositis, and the therapeutic effect is better than Ecdysterone administered on its own.

### 2.2. Ecdysterone-Paeonol Alleviates Inflammatory Cytokines Secretion by Inhibiting the NF-κB Pathway

**Figure 4:**
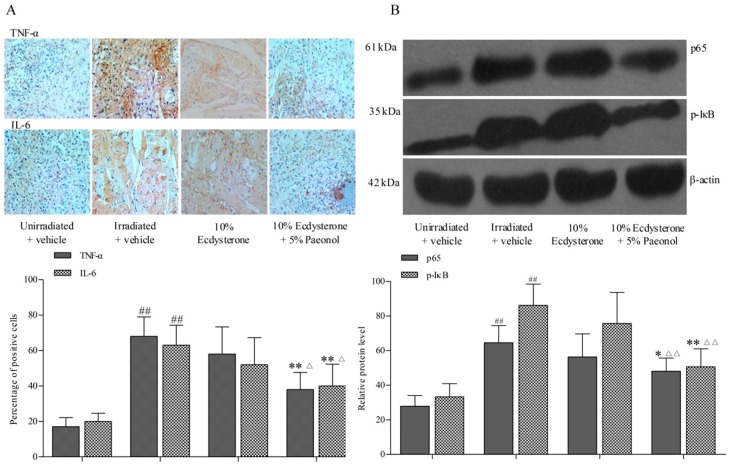
Immunostaining and Western blotting results after 12 days of treatment: (**A**) Representative images (above) are shown to demonstrate the immunostaining observed in the tongue sections for each of the treatment groups. The unirradiated + vehicle, irradiated + vehicle, 10% Ecdysterone, and 10% Ecdysterone + 5% Paeonol groups (^##^*p* < 0.01 vs. the unirradiated + vehicle group; **p < 0.01 vs. the irradiated + vehicle group; ^△^*p* < 0.05 vs. the 10% Ecdysterone group; ×100; *n* = 6 rats). (**B**) Representative p65 (64 kDa), - p-IκB (35 kDa) and β-actin loading control (43 kDa), showing expression and average relative protein levels in each group (^##^*p* < 0.01 vs. the unirradiated + vehicle group; *p < 0.05, **p < 0.01 vs. the irradiated + vehicle group; ^△△^*p* < 0.01 vs. the 10% Ecdysterone group; *n* = 6 rats).

The therapeutic effect of 10% Ecdysterone + 5% Paeonol is better than Ecdysterone administered on its own. The results that expressions of TNF-α and IL-6, two representative inflammatory cytokines, were detected were explored more deeply using immunohistochemistry assay. The expression of the 10% Ecdysterone and the irradiated + vehicle group were higher than the unirradiated + vehicle group. Even though the expression of 10% Ecdysterone was lower than the irradiated + vehicle group, there was no significantly difference. However, 10% Ecdysterone + 5% Paeonol reduced the expression of inflammatory cytokines compared with the unirradiated + vehicle group (Figure 4A). Meanwhile, the results of Western blotting assay indicated that the NF-κB signaling pathways were significantly down-regulated after 10% Ecdysterone + 5% Paeonol treatment (Figure 4B).

These results suggested that 10% Ecdysterone + 5% Paeonol reduced inflammatory cytokines secretion by down-regulating the NF-κB signaling pathway. It is Paeonol that makes up for the deficiency in the treatment of RIOM compared with Ecdysterone treatment alone.

### 2.3. Drug Targeting and Network Construction

Ecdysterone and Paeonol, two compounds isolated from traditional Chinese medicine herbs, might target multiple proteins. In order to probe the binding of their targets, SysDT methods were applied [12]. In the results shown in Table 1, 19 predicted targets associated with apoptosis, proliferation, inflammation, and wound healing, which play important roles in the development of RIOM, are screened out. For instance, Glycogen synthase kinase-3 beta (GSK3 β) is a negative regulator of apoptotic process protein [13]. Prothrombin (F2) is a positive regulator of cell growth and a negative regulator of cytokines production involved in inflammatory response protein [14,15]. Hematopoietic cell protein-tyrosine phosphatase 70Z-PEP (PTPN22) is a negative regulator of interleukin-6 secretion and the NIK/NF-kappaB signaling way [16]. Plectin (PLEC) is a wound healing regulator [17].

**Figure 5:**
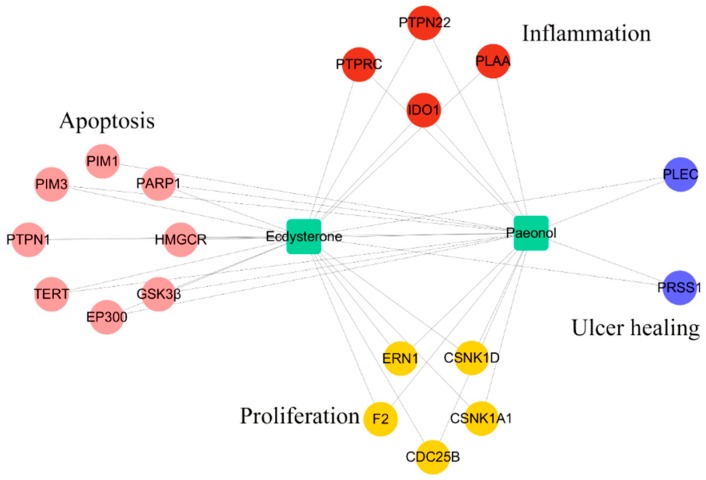
Compound−target (C–T) network constructed by linking Ecdysterone-Paeonol with its potential targets.

To uncover the synergistic effects of Ecdysterone-Paeonol on multiple targets in the treatment of radiation-induced oral mucositis, as well as to assess their mechanisms, a compound−target (C–T) network, as shown in Figure 5, was constructed using detailed information of the targets provided in Table 1. All of these results indicated that Ecdysterone-Paeonol affected multiple targets and showed a synergistic effect [18,19,20].

### 2.4. Molecular Docking Simulations

**Figure 6:**
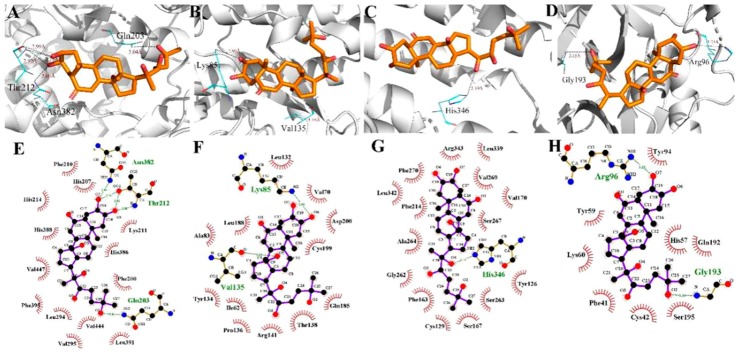
Detailed view of the binding interaction between Ecdysterone and four representative receptors. (**A**,**E**) F2-Prothrombin. (**B**,**F**) GSK3B-Glycogen synthase kinase-3 beta. (**C**,**G**) IDO1-Indoleamine 2,3-dioxygenase. (**D**,**H**) PRSS1-Trypsin-1.

**Figure 7:**
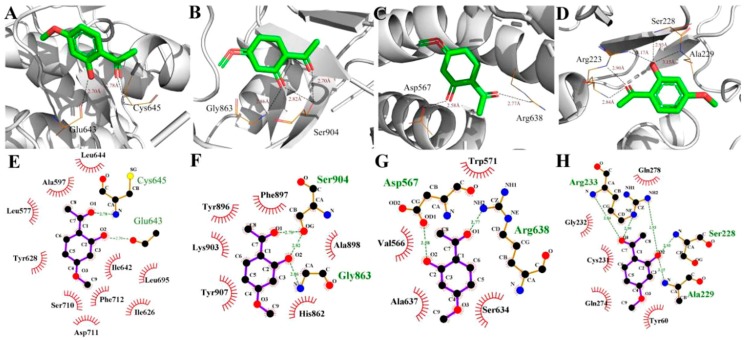
Detailed view of the binding interaction between Paeonol and four representative receptors. (**A**,**E**) ERN1-Serine/threonine-protein kinase/endoribonuclease IRE1. (**B**,**F**) PARP1-Poly [ADP-ribose] polymerase-1. (**C**,**G**) PLEC-Plectin. (**D**,**H**) PTPN22-Trypsin-1.

**Figure 8:**
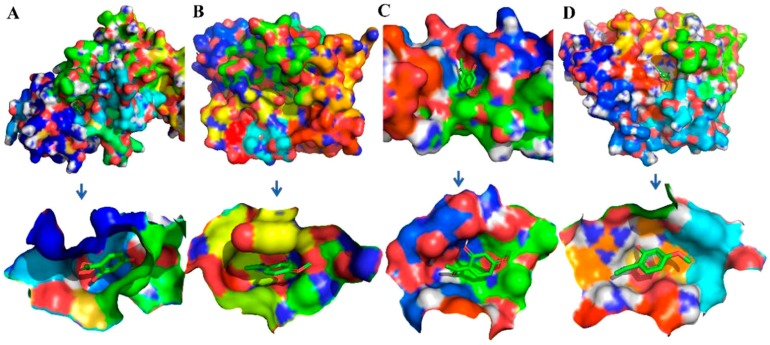
Binding cavity snapshots of Paeonol and four representative receptors. (**A**) ERN1-Serine/threonine-protein kinase/endoribonuclease IRE1. (**B**) PARP1-Poly (ADP-ribose) polymerase-1. (**C**) PLEC-Plectin. (**D**) PTPN22-Trypsin-1.

**Figure 9:**
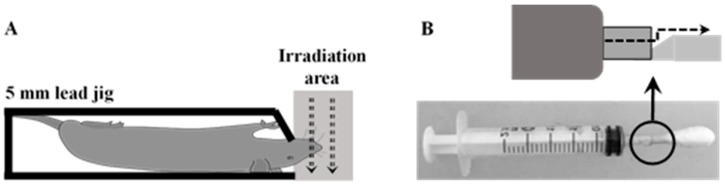
Radiation shielding materials box, oral administration device detail: (**A**) 5 mm lead jig allows for cranium irradiation. The gray dotted area indicates the irradiated area. (**B**) In-house built oral device with the detail shown above. A 2.5 mL syringe was repurposed. A groove was created to allow the solution to flow from the syringe into the cotton swab. The dotted line indicates the flow direction.

To evaluate the reliability of Ecdysterone and Paeonol and their target interactions, we simulated four representative targets for each compound with the AutoDock approach. The binding cavity snapshots are depicted in Figure 6 and Figure 7. The detailed view of the two-dimensional ligand interaction between Ecdysterone and Paeonol and four representative receptors as shown in Figure 8 and Figure 9.

**Figure 10:**
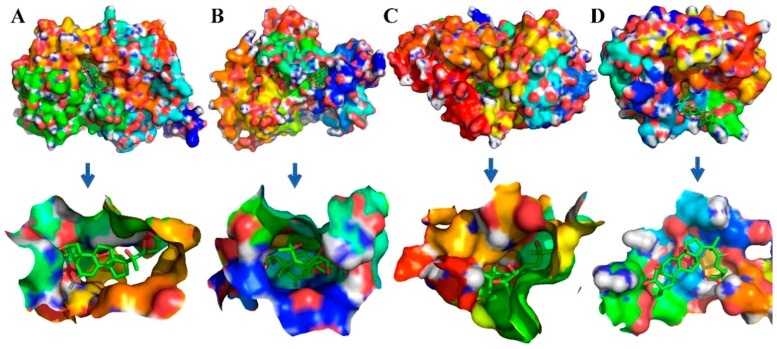
Binding cavity snapshots of Ecdysterone and four representative receptors. (**A**) F2-Prothrombin. (**B**) GSK3B-Glycogen synthase kinase-3 beta. (**C**) IDO1-Indoleamine 2,3-dioxygenase. (**D**) PRSS1-Trypsin-1.

As we can see in Figure 8 and Figure 10, eight receptors within the binding cavity are stable during the course of the molecular dynamics simulation, suggesting that the docking model was reliable. In addition, the results of the two-dimensional ligand interaction in Figure 6 and Figure 7 demonstrate that Ecdysterone and Paeonol anchor into a hydrophobic pocket, which is formed by different residues. All of these demonstrate that the ligand fits well with the binding site of the receptors, and the complex may become more stable after a longer time of molecule docking simulations [18].

### 2.5. Binding Free Energy Analysis

Binding free energy reflect the binding affinity between the compounds and the target proteins. We chose four target proteins of Ecdysterone and Paeonol as being representative. As we can see from the details in Table 2, free energies (−4.14 to −9.43 kcal/mol) indicated that two compounds showed high binding affinities to their targets and presented a high correlation with the bio-activities of Ecdysterone-Paeonol and the targets.

## 3. Discussion

Radiation-induced oral mucositis (RIOM), a normal oral cavity tissue injury resulting from radiotherapy and a huge challenge for radiation oncologists, represents an influential factor in cancer patients’ accepted radiation therapy, especially in head and neck cancer patients [3,21]. According to incomplete statistics, RIOM treatment adds an economic cost increase of up to 17,000.00 USD for every head and neck cancer patient undergoing radiation therapy [22], which is a huge economic burden. Fortunately, many companies have an interest in developing interventions for RIOM. A range of compounds are in pre-clinical or clinical development, such as Mammalian target of rapamycin (mTOR) inhibitors and recombinant protein. Even so, the management of mucositis is still austere [23].

In previous research, we discovered that Ecdysterone accelerates the healing of radiation-induced oral mucositis in rats by increasing matrix cell proliferation. These findings position Ecdysterone as a potential treatment candidate for oral mucositis in the clinic. However, after deeper research we found that, although Ecdysterone has an anti-inflammatory effect, the effect is only weak. Pro-inflammatory cytokines such as TNF-α and IL-6 were lower than in the modeling rats, but there was no significantly difference. These pro-inflammatory factors increase the vascular permeability and recruit more inflammatory cells. With the accumulation of pro-inflammatory cytokines and their negative feedback regulation, inflammation will continuously develop [24,25].

Mechanism is the guiding principle for increasing the efficacy of any drug. A good therapeutic effect is the end result that drug workers desire. In previous research, we only found the advantages of Ecdysterone [8]. Subsequently, as the research went deeper, we found the weaknesses of Ecdysterone. Therefore, according to the discovered mechanism in previous research, and in order to improve the therapeutic effects on RIOM, we had to choose a suitable drug to play a supplemental role. Therefore, in the present study, we chose a suitable anti-inflammatory drug and demonstrated that Ecdysterone-Paeonol reduced the accumulation of pro-inflammatory cytokines by down-regulating the NF-κB signaling pathway. Paeonol made up for the deficiency in the treatment of RIOM compared with Ecdysterone oral cavity direct administrated on its own. In addition, Paeonol has a good transdermal absorption, which is suitable for oral direct administration. Moreover, Paeonol has been applied for its anti-inflammatory, antioxidant, and antibacterial properties for a long time in traditional Chinese medicine [26,27,28].

Systems pharmacology dissection indicated that the Ecdysterone-Paeonol targeted multiple-targets in the treatment of RIOM. All of these targets affected different functions such apoptosis, proliferation, inflammation, and wound healing, which present different functions in the course of the pathological development of RIOM. These two compounds works synergistically in improving the therapeutic effect. Meanwhile, the approach developed in this work offers a new understanding of the action mechanisms, which will promote the development and application of simple Chinese medicine monomer formulas [29,30].

However, in this research we only used Paeonol as the combination drug [31]. Even though the therapeutic effect was better than Ecdysterone administered on its own, the ratio test should be evaluated in the future, which might be another way to enhance the therapeutic effect. Moreover, there are a large number of anti-inflammatory drugs in traditional Chinese medicine, so whether Paeonol is the best one to use should also be researched in the future.

## 4. Material and Methods

### 4.1. Animals

Male Sprague Dawley (SD) rats (180–220 g, 6–8 weeks) were provided by the Experimental Animal Center of Anhui Medical University (Hefei, China). Animal experiments were approved by the Institutional Animal Care and Use Committee of Hefei University of Technology (IACUC, Permit No.1809151121, date 09/01/2018).

### 4.2. Rats’ Living and Feeding Conditions

Living conditions before and after irradiation: plastic cages filled with large sawdust on the bottom, 20 ± 2 °C, 60 ± 10% humidity, 12 h light-dark cycle with lights on at 7:00 a.m.

Feeding conditions before irradiation: Free access to tap water and a normal diet.

Feeding conditions after irradiation: Free access to sterilized (not tap) water and sterilized food (sterilized with ultraviolet irradiation).

### 4.3. Radiation-Induced Oral Mucositis Rat Model

Rats were randomly divided into 4 groups including 1) unirradiated + vehicle, 2) irradiated + vehicle, 3) 10% Ecdysterone, and 4) 10% Ecdysterone + 5% Paeonol. There were 12 rats in each group. Before irradiation, the rats were anesthetized. Subsequently, they were kept in the 5 mm lead jig and exposed to a single dose of 20 Gy X-rays (Varian Medical System, 6 MV, dose rate: 250 cGy/min) (Figure 9A), in accordance with previous research [32]. After irradiation, the rats were placed in separate boxes in order to prevent suffocation death caused by different anesthesia recovery times. Once all the rats were awake, the rats in different groups were gathered together and fed according to the conditions after irradiation mentioned above.

### 4.4. Treatment Administration

10% Ecdysterone and 5% Paeonol was dissolved in 0.5% CMC-na, and ultrasound was used to keep the compound in solution prior to administration. An in-house constructed syringe device was applied for the administration (Figure 9B). Rats in each group were treated with vehicle or compounds three times daily, 2 mL each time (at 9:00 am, 2:00 pm, and 6:00 pm) [32]. After 12 days of treatment, some tongue samples in each group were harvested for direct observation, immunohistochemistry, and Western blotting analyses. During this course of treatment, rats were anesthetized and tongues were pulled for visual scoring observation every 2nd day, according to the “visual scoring assessment of oral mucositis” in previous research [32], and average weight was taken daily. Visual scoring assessment was done blind by the same three people in real time. Subsequently, the remaining rats were treated and evaluated for the total course of the treatment until the RIOM needed only basic rehabilitation.

### 4.5. Immunohistochemistry Assay

To examine inflammatory cytokines, immunohistochemistry was performed on damaged areas of tongues harvested post-treatment, as described previously [32]. The antibodies used in this study included primary antibodies for different leukocyte markers, including monoclonal rabbit-anti-rat TNF-α (diluted 1:100) (Abcam Antibodies, Cambridge, UK), rabbit-anti-ra IL-6 (diluted 1:100) (Abcam Antibodies, Cambridge, UK), and biotinylated secondary goat antibody IgG (1:100, Invitrogen, Carlsbad, USA).

### 4.6. Western Blotting Assay for NF-κB Pathway

We performed protein extraction and Western blotting analyses as previously described [32]. In the test, we used antibodies including monoclonal rabbit-anti-rat Ras (1:1000, Bioss Biotechnology, Beijing, China), rabbit-anti-rat p65 (1:1000, Bioss Biotechnology), rabbit-anti-rat p-IκB (1:500, Bioss Biotechnology, Beijing, China), and rabbit-anti-rat β-actin (1:10000, Bioss Biotechnology, Beijing, China). Goat-anti-rabbit IgG (Bioss Biotechnology, Beijing, China) was used as a secondary antibody. Gray-scale images and quantification were analyzed with Image-J software (National Institutes of Health, Bethesda, MD, USA).

### 4.7. Target Fishing and Network Construction

To obtain the molecular targets of Ecdysterone-Paeonol, SysDT methods were proposed, which efficiently integrated large scale information on targets [12]. Additionally, we characterized a compound−target (C−T) network to explore the biological effects of Ecdysterone-Paeonol on different function proteins in the treatment of RIOM [33].

### 4.8. Molecular Docking

Presently, to calculate the interactions between Ecdysterone-Paeonol and its targets, four important predicted and approved targets associated with apoptosis, proliferation, inflammation, and wound healing were selected for docking. By using the molecular docking program AutoDock (Scripps Research Institute, San Diego, USA), simulations were performed [34]. The unit uses an X-ray crystal structure of eight targets (F2, GSK3B, IDO1, PRSS1, ERN1, PARP1, Plectin, PTPN22), taken from the RCSB Protein Data Bank. Each protein was prepared using methods such as adding polar hydrogens, partial charges, and defining the rotatable bonds. The files were then input into AutoDock. Finally, the results were analyzed using the AutoDock Tools [35].

### 4.9. Statistical Analysis

All statistics are presented as mean ± standard deviation (std. dev.). A Student’s t-test was used for statistical comparisons. Ulcer size measurements are given as mean ± standard error of the mean (SEM). A one-way ANOVA was done for the multiple groups.

## 5. Conclusions

In summary, the Ecdysterone-Paeonol combination drug alleviated the development of radiation-induced oral mucositis and reduced the total basic recovery days. As the guiding principle for increasing the efficacy of the drug, mechanism allowed investigators to demonstrate the weakness of Ecdysterone and made up for the deficiency through the combination administration method. Paeonol presents a suitable supplement drug. The Ecdysterone-Paeonol combination drug reduces pro-inflammatory cytokines production by inhibiting the NF-κB pathways. Systems pharmacology dissection results uncovered the detailed molecular mechanism and multiple targets of Ecdysterone-Paeonol. These findings position Ecdysterone-Paeonol as a potential treatment candidate. The development of twin medicines or multiple targets drug exploitation will be the future direction of our research group.

## Figures and Tables

**Table 1:** Information of Targets of Ecdysterone and Paeonol.

Function	Target	Common Name	Uniprot ID
Apoptosis	Telomerase reverse transcriptase	TERT	O14746
	HMG-CoA reductase	HMGCR	P04035
	Poly [ADP-ribose] polymerase-1	PARP1	P09874
	Protein-tyrosine phosphatase 1B	PTPN1	P18031
	Glycogen synthase kinase-3 beta	GSK3β	P49841
	Histone acetyltransferase p300	EP300	Q09472
	Serine/threonine-protein kinase PIM1	PIM1	P11309
	Serine/threonine-protein kinase PIM3	PIM3	Q86V86
Proliferation	Serine/threonine-protein kinase/endoribonuclease IRE1	ERN1	O75460
	Prothrombin	F2	P00734
	Dual specificity phosphatase Cdc25B	CDC25B	P30305
	Casein kinase I alpha	CSNK1A1	P48729
	Casein kinase I delta	CSNK1D	P48730
Inflammation	Leukocyte common antigen	PTPRC	P08575
	Indoleamine 2,3-dioxygenase	IDO1	P14902
	Phospholipase A-2-activating protein	PLAA	Q9Y263
	Hematopoietic cell protein-tyrosine phosphatase 70Z-PEP	PTPN22	Q9Y2R2
Wound healing	Trypsin-1	PRSS1	P07477
	Plectin	PLEC	Q15149

**Table 2:** Binding energy of Ecdysterone and Paeonol between four representative receptors.

Compound	Target	Binding Energy (kcal/mol)
Ecdysterone	PRSS1	−4.14
Ecdysterone	F2	−6.92
Ecdysterone	GSK3B	−7.11
Ecdysterone	IDO1	−9.43
Paeonol	Plectin	−4.45
Paeonol	ERN1	−5.12
Paeonol	PARP1	−5.45
Paeonol	PTPN22	−5.61
